# Prevalence, antimicrobial resistance pattern, and associated factors of *Salmonella* and *Shigella* among under five diarrheic children attending public health facilities in Debre Markos town, Northwest Ethiopia

**DOI:** 10.3389/fpubh.2023.1114223

**Published:** 2023-02-06

**Authors:** Mesfin Dessale, Getachew Mengistu, Hylemariam Mihiretie Mengist

**Affiliations:** ^1^Department of Medical Laboratory Sciences, College of Medicine and Health Sciences, Debre Markos University, Debre Markos, Ethiopia; ^2^Gidan Health Center, Gidan Woreda Health Office, Gidan, Ethiopia

**Keywords:** prevalence, antimicrobial resistance pattern, *Salmonella*, *Shigella*, under-five children, Debre Markos, Ethiopia

## Abstract

**Background:**

Under five children are at risk of diarrhea-associated morbidity and mortality. *Salmonella* and *Shigella* are major causes of diarrhea in under-five children, especially in developing countries. This study aimed to assess the prevalence, antimicrobial resistance pattern, and associated factors of *Salmonella* and *Shigella* among under-five diarrheic children in Debre Markos town public health facilities.

**Methods:**

A cross-sectional study was conducted at public health facilities in Debre Markos town using a consecutive convenient sampling technique. Data on socio-demographic and associated factors were collected using a structured questionnaire. *Salmonella* serovars and *Shigella* species were identified using MacConkey, Xylose Lysine Deoxycholate, *Salmonella Shigella* agar, and biochemical tests. The antimicrobial resistance pattern was determined by using the modified Kirby-Bauer disk diffusion technique.

**Results:**

The overall prevalence of *Salmonella* and *Shigella* was 11.7% (26/222; 95% CI = 7.2–17.5%). Isolated *Salmonella* serovars showed a higher rate of resistance (85.7%, 6/7) for both Ampicillin and Amoxicillin/Clavulanic acid while *Shigella* isolates showed a higher resistance rate to Amoxicillin/Clavulanic acid (78.9%, 15/19) and Ampicillin (73.7%, 14/19). The overall multidrug resistance (MDR) rate of *Salmonella* and *Shigella* isolates was 88.5% (23/26). Parent/guardian educational status ≤ elementary school (AOR = 3.783; 95% CI = 1.28–11.19; *P* = 0.016), presence of two or more under-five children in the family (AOR = 8.999; 95% CI = 2.93–27.69; *P* < 0.001), unimproved source of drinking water (AOR = 5.010; 95% CI = 1.56–16.10; *P* = 0.007), the habit of storing cooked foods for later use (AOR = 3.199; 95% CI = 1.07–9.54; *P* = 0.037), attendance of the child at social gatherings (AOR = 5.387; 95% CI = 1.78–16.35; *P* = 0.003), and infrequent child fingernail trimming (every ≥ 2 weeks; AOR = 4.693; 95% CI = 1.47–14.94; *P* = 0.009) showed statistically significant association with the prevalence of culture-confirmed *Salmonella* and *Shigella* isolates.

**Conclusion:**

The prevalence of culture-confirmed *Salmonella* and *Shigella* isolates was significantly high in the study area. *Salmonella* and *Shigella* isolates exhibited a high rate of MDR pattern. Parent/guardian education level below the elementary school, the presence of two or more under-five children in the family, using unimproved water source, a habit of storing cooked food, and infrequent fingernail trimming were independent predictors of culture-confirmed *Salmonella* and *Shigella*. Therefore, besides public health measures, regular surveillance of the prevalence and antimicrobial resistance pattern of *Salmonella* and *Shigella* should be routinely practiced in the study setting.

## Introduction

According to World Health Organization (WHO) definition, diarrhea is the passage of three or more abnormal stools (such as loose, bloody, or liquid stool) per day (1). Diarrhea can be mainly caused by protozoa, viruses, and bacteria. Most prevalent diarrheal cases are caused by bacteria (2) and *Clostridium botulinum, Campylobacter jejuni, Vibro cholerae, Escherchia coli, Staphylococcus aureus, Salmonella* serovars, and *Shigella* species are the predominant causative agents of food poisoning, enteric fever, and gastroenteritis (3, 4).

*Salmonella* serovars and *Shigella* species belong to the family *Enterobacteriaceae*, and they are among the predominant causative agents of food poisoning, enteric fever, and gastroenteritis. They are responsible for the high prevalence of under-five diarrhea, especially in developing countries owing to lack of access to clean water and poor hygiene (3, 4). Both *Salmonella* serovars and *Shigella* species remain to be the foremost cause of acute diarrhea in resource-limited countries and cause serious health problems (5).

Salmonellosis is caused by the genus *Salmonella* which has two species (*Salmonella Enterica* and *Salmonella Bongori*) and over 2,500 serotypes. *Salmonella* is gram-negative, facultatively anaerobe, rod-shaped, motile, non-lactose fermenting, oxidase-negative, and non-spore-forming bacteria. *Salmonella* is the leading cause of food-borne infections throughout the world. The route of transmission of *Salmonella* is feco-oral mainly with contaminated food and water. Once entered, the bacterium attaches to the small intestinal mucosa and invades M cells with subsequent multiplication in the cytoplasmic vacuole. Then, the bacterium will be released into the bloodstream and lymphatic circulation. After 12–72 h of infection, fever, diarrhea, abdominal cramp, and vomiting will appear (6). The infection is more serious in geriatric (above 60 years) and pediatric (under-5 years) patients (7, 8). *Salmonella* serovars cause diseases ranging from self-limiting gastroenteritis to more severe systemic typhoid fever (8, 9). More than 27 million infections and 200,000 deaths occur each year globally due to *Salmonella* infection (8).

Shigellosis is caused by the genus *Shigella* which has four species (*Shigella dysenteriae, Shigella sonnei, Shigella boydii*, and *Shigella flexneri*). Genus *Shigella* contains gram-negative, facultatively anaerobe, rod-shaped, non-motile, non-lactose fermenting, bile salt resistant, oxidase-negative, and non-spore-forming bacteria. The route of transmission for *Shigella* is also feco-oral mainly through contaminated hands and less commonly through contaminated water and food. As few as 100–200 bacteria can establish shigellosis (8, 10).

*Shigella* species are acid-tolerant and can reach the intestine by passing through the stomach. They produce Shiga toxin and disrupt protein synthesis in mucosal epithelial cells, causing bacillary dysentery, and watery or bloody diarrhea. *Shigella* are limited to the human and animal intestinal tract only. Personal and environmental hygiene practices are directly related to both the incidence and spread of the disease (10). Although it is a global problem, shigellosis is more prevalent in developing countries (10, 11).

Different factors may contribute to the transmission and spread of bacterial etiologies of diarrhea in under-five children. These factors could be personal and/or environmental factors, behavioral and hygiene-related factors, socio-demographic factors, or clinical factors (12–20).

Although it is easily preventable, diarrhea is not only the cause of mortality and morbidity in under-five children but also a reason for the loss of a huge amount of money. A systematic review from 2006 to 2018 showed that a weighted mean per episode expenditure was US$ 216.36 and 52.16 for inpatient and outpatient children, respectively in low and middle-income countries (21). A study conducted in Ethiopia on under-five children reported that the respective mean per episode expenditure was 79 and 6 US$ for inpatient and outpatient children in 2013 (22). The highest burden of diarrhea due to *Salmonella* serovars and *Shigella* species in under-five children occurs in sub-Saharan Africa (4, 23–25). The burden of *Salmonella* and *Shigella* among under-five children had different magnitudes in different parts of Ethiopia. The prevalence of *Salmonella* serovars *and Shigella* species was high in Northwest Ethiopia (17.3%) (26).

Antimicrobial resistance is a big global burden associated with the increasing risk of economic burden, mortality, and morbidity in developing countries. These countries are victims of antimicrobial resistance because of unnecessary consumption, compromised drug quality, lack of continuous surveillance, poverty, and antimicrobial misuse in cattle and poultry feed (27). In developing countries, the increasing emergence of antimicrobial-resistant *Salmonella* serovars and *Shigella* species to commonly prescribed antimicrobials such as chloramphenicol, ampicillin, tetracycline, and co-trimoxazole has been documented in recent years (18, 20, 28). Therefore, continuous study is important to help solve the problem.

In Ethiopia, the high prevalence of salmonellosis and shigellosis in under-five children is associated with different factors including the lower level of maternal education status, low family economic status, improper disposal of waste, poor handwashing practice, improper disposal of child's feces, low access to clean potable water, and the absence/irregular use of toilet (19, 20). According to data from the central statistics agency, the accessibility of improved drinking water sources and toilet facilities in rural households of Ethiopia was only 57 and 4%, respectively (29). Around 88% of diarrhea-related death is estimated to be due to unsafe water, insufficient hygiene, and inadequate sanitation (30).

Children are highly vulnerable to *Salmonella* and *Shigella* infection due to immaturity of the immune system, and high contagiousness of the organisms with low infectious doses. *Salmonella* serovars and *Shigella* species that have been frequently isolated in clinical samples showed a varied resistance pattern for commonly prescribed antimicrobials which makes the treatment difficult (19, 20). In Ethiopia, a few studies have been conducted on the prevalence and antimicrobial resistance pattern of *Salmonella* serovars and *Shigella* species among under-five diarrheic children, especially in the study area. The main aim of this study was, therefore, to assess the prevalence, antimicrobial resistance pattern, and associated factors of *Salmonella* and *Shigella* among under-five diarrheic children in Debre Markos town public health facilities.

## Materials and methods

### Study area and setting

The study was conducted in public health facilities in Debre Markos town. Debre Markos is the capital of the East Gojjam zone which is 295 km Northwest of Addis Ababa, the capital city of Ethiopia, and 264 km Southeast of Bahir Dar, the capital of Amhara National Regional State. The geographical location of Debre Markos is 10°17′00′′ to 10°21′30′′ N Latitudes and 37°42′00′′ to 37°45′30′′ E longitudes and it has an elevation of 2,350–2,500 m above sea level (31). The city has 11 administrative kebeles and the population size is estimated to be 111,313 in 2018 (32) of which 51,358 are males. There are four public health centers [Gozamen Health Center (GHC), Debre Markos Health center (DMHC), Wuseta Health Center (WHC), and Hidase Health Center (HHC)] and one comprehensive specialized hospital, Debre Markos comprehensive specialized hospital (DMCSH) in the town.

### Study design and period

A cross-sectional study was conducted from June 02 to August 29, 2022.

### Source population

All under-five diarrheic children seeking medical service in Debre Markos town public health facilities were the source population.

### Study population

All eligible under-five diarrheic children seeking medical service at public health facilities in Debre Markos town during the study period were the study population.

### Eligibility

All diarrheic under-five children ≥6 months of age were included in the study while children who took antimicrobials in the past 2 weeks before specimen collection and those treated for the current diarrhea attack were excluded from the study.

### Sample size determination and sampling technique

The sample size was calculated by using a single population proportion formula based on the assumption of a 95% confidence interval (Zα/2 = 1.96), 5% margin of error, and a prevalence of 17.45% from a previous study in Arba Minch, Ethiopia (18). Accordingly,


$$\begin{array}{l} n=\frac{{\left(Z_{\frac{\alpha }{2}}\right)}^{2}*\;P\left(1-P\right)}{d^{2}} \end{array}$$

p = previous prevalence of *Salmonella* and *Shigella*.
d = margin of error.
*n* = (1.96)^2^* (0.1745 * 0.0.8255) ÷ 0.0025.
*n* = 222.

**Figure 1:**
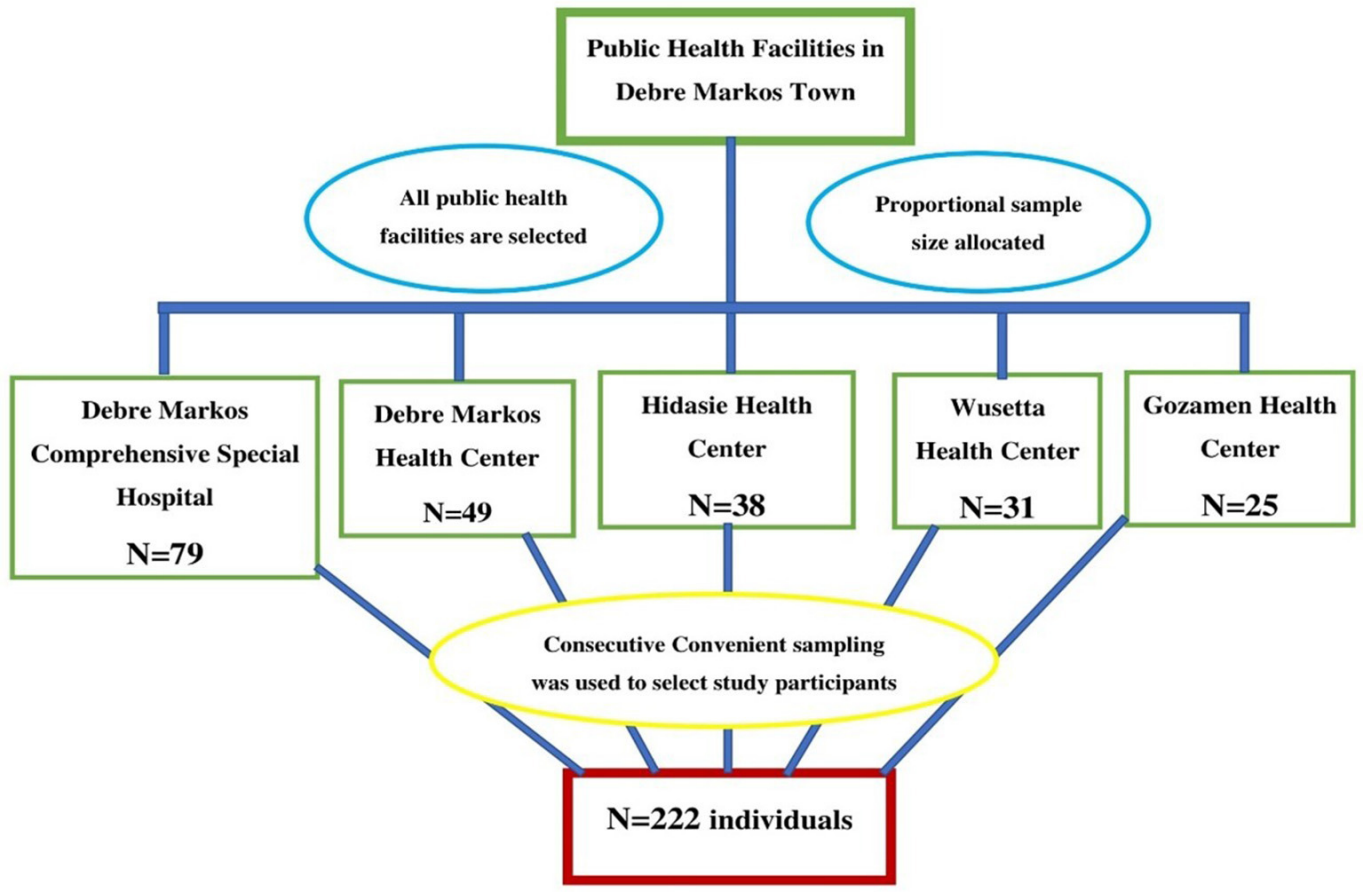
Proportional allocation of study participants among public health facilities in Debre Markos town, 2022.

Finally, a total of 222 study participants were enrolled by using a consecutive convenient sampling technique. Study participants were allocated proportionally for all public health facilities in the study area based on 2021/2022 (2014 E.C) fiscal year second quarter under-five children's diarrhea report. The 2014 E.C second quarter diarrhea report of DMCSH, DMHC, HHC, WHC, and GHC was 617, 381, 294, 239, and 189, respectively. So, 79, 49, 38, 31, and 25 samples were taken from DMCSH, DMHC, HHC, WHC, and GHC, respectively (Figure 1).

### Data collection

Data on socio-demographic, clinical, and hygiene-related factors were collected through face-to-face interviews with a structured questionnaire adapted from Ethiopian demographic health survey and other previous studies (29, 33). Parents/guardians were interviewed in the Amharic language (local language) by trained nurses. The nutritional status of the study participants was assessed by measuring their mid-upper arm circumference (MUAC).

### Specimen collection and transportation

Stool specimens were collected, handled, and properly stored by trained laboratory professionals. A clean and properly labeled stool specimen container was given to parents/guardians after explaining how to collect the stool specimen. A single stool specimen about 1 gm or 1 ml (one teaspoonful in case of loose stools) was collected from each study participant by using a screwcap container (6). Then, it was placed into a Cary-Blair transport medium and was transported to the DMCSH microbiology laboratory for analysis within 3 h of collection.

### Bacterial isolation and identification

By using a sterile wire loop, the stool sample was inoculated into MacConkey agar (Oxoid Ltd) and incubated at 37°C aerobically for 24 h. Then, non-lactose fermenting colorless/pale colonies were inoculated into Xylose Lysine Deoxycholate (XLD) agar (Oxoid Ltd) and *Salmonella Shigella* (SS) agar (Oxoid Ltd) and incubated aerobically at 37°C for 24 h. Finally, pink to red colonies on XLD agar and colorless colonies on SS agar with or without black centers were further analyzed with biochemical tests for the identification of *Salmonella* serovars and *Shigella* species (6).

Biochemical tests were performed on pink to red colonies from XLD agar and/or colorless colonies from SS agar with or without black centers for the identification of the *Salmonella* serovars and *Shigella* species. Bacteria were identified by using triple sugar iron agar (TSI; H_2_S and gas production, carbohydrate fermentation), motility test, indole test, citrate utilization test, urea hydrolysis test, lysine decarboxylation (LDC), mannitol fermentation, and oxidase tests.

### Antimicrobial susceptibility testing

The antimicrobial resistance pattern of bacteria was determined by using the modified Kirby–Bauer disk diffusion technique on Mueller–Hinton agar (MHA; Oxoid Ltd) according to the Clinical and Laboratory Standards Institute (CLSI 2021) guideline (34). The inoculum was prepared by suspending 4–5 isolated pure colonies in 5 ml of nutrient broth and incubated at 37°C for 30 min. The turbidity of the suspension was adjusted with 0.5 McFarland's standard. A sterile cotton swab was used to inoculate the organism from the broth into the MHA plates uniformly. Antimicrobial disks; Ampicillin (10 μg), Amoxicillin-clavulanic acid (20/10 μg), Azithromycin (15 μg), Ceftazidime (30 μg), Ceftriaxone (30 μg), Chloramphenicol (30 μg), Ciprofloxacin (5 μg), Doxycycline (30 μg), Tetracycline (30 μg), and Trimethoprim-sulfamethoxazole (1.25/23.75 μg; Oxoid Ltd) were tested for both *Salmonella* serovars and *Shigella* species (34). Antimicrobial disks were placed and incubated at 35–37°C overnight. The diameter of the zone of inhibition was measured and interpreted based on CLSI 2021 guidelines (34). The result of each antimicrobial test was interpreted as sensitive, intermediate, or resistant (34). Isolates were reported as MDR when resistance to at least one antimicrobial agent in three or more antimicrobial categories was observed (35).

### Data quality management

The questionnaire was first prepared in English language and translated into the local language (Amharic). Then, it was translated back into the English language to assure consistency. The questionnaire was validated through conducting a pre-test on 10% of the total sample size (23 clients) at Lumamie primary hospital and modifications were made accordingly.

Data collectors (nurses) and laboratory professionals were trained for 1 day and Standard Operating Procedures (SOPs), as well as manufacturer's instructions, were strictly followed during specimen collection, transportation, and processing. The collected stool specimens were transported with maximum care using a cold chain and transport medium and tested within 3 h. Stool specimens with insufficient volume or contaminated with urine, soil, or water were rejected. The dry hot oven sterilization method was applied to test tubes, cotton swabs, and Petri dishes. The sterility of culture media and biochemical tests for each batch was checked by incubating 5% of the prepared media without inoculation for 24 h at 37°C.

*E. coli* ATCC^®^ 25922, *P. aeruginosa* ATCC^®^ 27853^e^, *S. typhimurium* ATCC 14028 (34), and *S. flexnerii* ATCC 12022 (36) were reference strains used to check the performance of the culture media, biochemical tests, and antimicrobial disks. Moreover, quality control strains were tested for growth, resistance pattern, and biochemical tests parallel with stool samples to assess the validity of the test procedure. The expiry date of all reagents, supplies and antimicrobial disks was also checked.

Standard laboratory data management policies and protocols were followed during result interpretation, documentation, and reporting. The results were recorded carefully using a laboratory result registration sheet. Generally, close supervision was undertaken seriously, and questionnaires were checked for completeness during and after data collection. Each data was checked for completeness before processing.

### Data processing and analysis

Cleaned and coded data were entered into EpiData version 4.6 and exported to SPSS version 25.0 for statistical analysis. Descriptive statistics were presented using tables, graphs, and percentages. Binary logistic regression models were performed to determine the presence of significant association between predictors and the dependent variable. Predictor variables that had a significant association in bivariable logistic regression (*P* ≤ 0.25) were fitted to multivariable backward stepwise logistic regression for final analysis and variables with *P*-value ≤ 0.05 were considered statistically significant. The model fitness was checked using Hosmer and Lemeshow test. The strength of association was measured using an odds ratio at 95% CI. The antimicrobial resistance pattern of *Salmonella* and *Shigella* isolates was analyzed using WHONET software version 22.8.23. MUAC measurement value was used to classify the nutritional status of the participants. Participants were classified as under-nutrition when their MUAC was ≤ 12.5 cm (37).

### Ethical considerations

Ethical clearance was obtained from Debre Markos University College of Health sciences before the actual data collection with letter number HSC/R/C/Ser/PG/Co/195/11/14 and Amhara public health institute with a letter numbered 03/1434. A supporting letter was written to the zonal health department from the college with letter numbered 196/11/14. Written permission to conduct the study was obtained from East Gojjam Zone health office and Debre Markos town health office. Then, a support letter was written to health institutions to obtain permission for the data collection process. Written informed consent was obtained from the parents/guardians of the study participants. Consent was taken from parents/guardians because our study participants were too young to volunteer to participate as well as to decide to withdraw from the study. Participation was only based on voluntarism and participants' parents/guardian were informed that they have the right to withdraw participants from the study at any time. Issues of confidentiality and anonymity were also maintained. The result of each study participant was reported to their respective clinician immediately for appropriate intervention.

### Operational definition

✓ Infrequent fingernail trimming: trimming fingernail in ≥2 weeks.
✓ Attendance at social gatherings: attending social gatherings within a week before the clinical onset of diarrhea.
✓ Unimproved source of drinking water: water not treated with chemicals before consumption.
✓ Poor house floor: when the house floor is not covered with plastic shed or ceramic.

## Results

### Socio-demographic characteristics of the study participants

A total of 222 study participants were enrolled in the study of which 95.5% (212/222) were urban dwellers and 52.7% (117/222) were males. The age of study participants ranged from 6 to 59 months with a median of 33 months (IQR = 30.25). About 75.7% (168/222) of parents/guardians were married and the family monthly income ranged from 1,500 to 12,500 Ethiopian Birr with a median of 8,000 Birr (IQR = 3,500; Table 1).

**Table 1:** Socio-demographic characteristics of the study participants in public health facilities in Debre Markos town, 2022.

**Variable**	**Variable category**	**Frequency**	**Percentage (%)**
Child sex	Male	117	52.7
	Female	105	47.3
Child age (in months)	6–12	39	17.6
	13–36	93	41.9
	37–59	90	40.5
Daycare (KG) attendance	Yes	63	28.4
	No	159	71.6
Parent/guardian age (in years)	< 29	99	44.6
	30–39	92	41.4
	>40	31	14.0
Residence area	Rural	10	4.5
	Urban	212	95.5
Parent/guardian educational level	≤ Elementary school	51	23.0
	≥Secondary school	171	77.0
Parent/guardian marital status	Divorced or widowed	54	24.3
	Married	168	75.7
Number of under-five children in the family	One	181	81.5
	Two and above	41	18.5
Family monthly income (in Birr)	< 4,500	31	14.0
	4,501–7,500	77	34.7
	7,501–10,000	89	40.1
	>10,001	25	11.3
Parent/guardian occupation	Civil servant	87	39.2
	Self-employed	87	39.2
	Housewife	48	21.6
Family size	2–4	138	62.2
	5–7	84	37.8

### Clinical characteristics of the study participants

Less than half (44.1%, 98/222) of the study participants were under-nutrition. The frequency of diarrhea per day ranged from 3 to 12 with a mean of 4.91 (SD ± 1.257). Family history of diarrhea in the past week was reported in 20.3% (45/222) of the study participants. Details are described in Table 2.

**Table 2:** Clinical characteristics of the study participants in public health facilities in Debre Markos town, 2022.

**Variable**	**Variable category**	**Frequency**	**Percentage (%)**
Previous history of child diarrhea in the last month	Yes	45	20.3
	No	177	79.7
Child nutritional status	Under-nutrition	98	44.1
	Normal	124	55.9
History of diarrhea within the family in the past week	Yes	45	20.3
	No	177	79.7
Parent/guardian history of the underline disease	Yes	10	4.5
	No	212	95.5
Presence of vomiting	Yes	114	51.4
	No	108	48.6
Presence of tenesmus	Yes	76	34.2
	No	146	65.8
Type of diarrhea	Bloody	38	17.1
	Mucoid	95	42.8
	Watery	89	40.1

### Hygiene-related characteristics of the study participants

Most of the study participants (93.7%, 208/222) had good house floor material. All respondents had a latrine and about half of them (51.4%, 114/222) had a handwashing facility near the latrine. One-third (33.3%, 74/222) of the study participants had a history of attendance at social gatherings within 7 days before disease onset. Around one-fifth (18.9%, 42/222) of the respondents had a travel history. The source of drinking water for most study participants (72.1%, 160/222) was improved (Table 3).

**Table 3:** Hygiene-related and behavioral characteristics of the study participants in public health facilities in Debre Markos town, 2022.

**Variable**	**Variable category**	**Frequency**	**Percentage (%)**
House floor material	Poor	14	6.3
	Good	208	93.7
Presence of hand washing facility near the latrine	Yes	114	51.4
	No	108	48.6
Parent/guardian hand washing habit after toilet	Sometimes or no	44	19.8
	Usually or always	178	80.2
General hand washing method	With water only	128	57.7
	With soap and water	94	42.3
Presence of a separate waste disposal system	Yes	136	61.3
	No	86	38.7
The habit of washing a child's hands after child defecation	Sometimes or no	52	23.4
	Usually or always	170	76.6
Parents/guardian habit of washing their hands after defecating the child	Sometimes or no	3	1.4
	Usually or always	219	98.6
Hand-washing habit before feeding the child	Sometimes or no	30	13.5
	Usually or always	192	86.5
Source of drinking water	Unimproved	62	27.9
	Improved	160	72.1
Use of a dipper (jug) to draw water from containers	Yes	206	92.8
	No	16	7.2
Use of narrow-necked containers for drinking water storage	Yes	217	97.7
	No	5	2.3
The habit of storing cooked food for later use	Sometimes or no	162	73
	Usually or always	60	27
The habit of storing trimming (leftover) food for later use	Sometimes or no	208	93.7
	Usually or always	14	6.3
Consumption of raw/uncooked food in the past week	Yes	44	19.8
	No	178	80.2
A habit of washing child food utensils before and after feeding the child	Sometimes or no	2	0.9
	Usually or always	220	99.1
Consumption of food from hotels, restaurants, or cafes	Yes	16	7.2
	No	206	92.8
Contact with a domestic animal in the past week	Yes	35	15.8
	No	187	84.2
Attendance at social gatherings in the past week	Yes	74	33.3
	No	148	66.7
Travel history of the child in the past week	Yes	42	18.9
	No	180	81.1
The time interval for fingernail trimming	Two weeks and above	72	32.4
	Below 2 weeks	150	67.6

### Prevalence of *Shigella* and *Salmonella* isolates

The overall prevalence of *Salmonella* and *Shigella* among the study participants was 11.7% (26/222; 95% CI = 7.2, 17.5%). Among these, the prevalence of *Salmonella* serovars, *S. dysenteriae*, and other *Shigella* species was 3.15% (7/222), 3.60% (8/222), and 4.95% (11/222), respectively.

**Figure 2:**
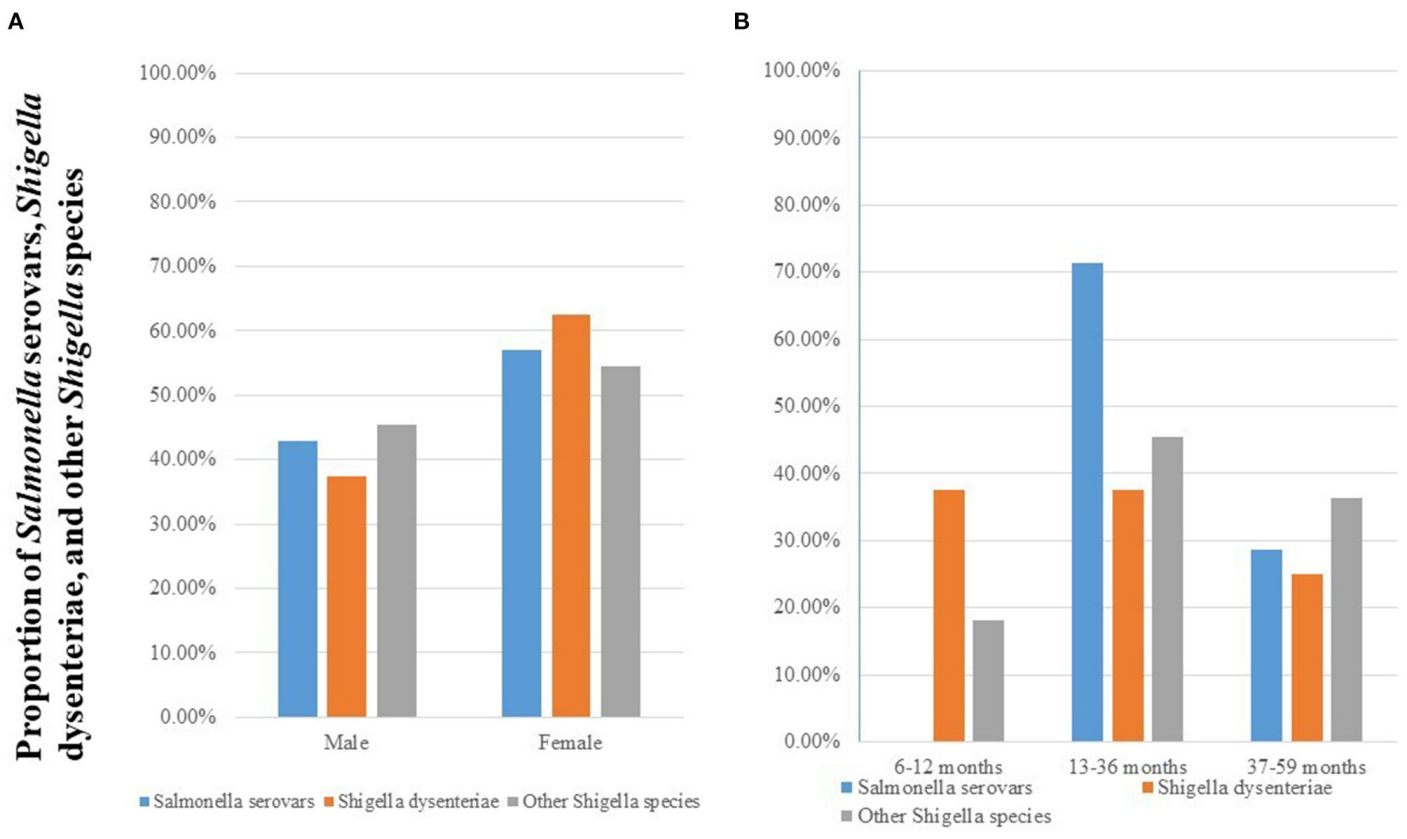
Prevalence of *Salmonella* serovars, *S. dysenteriae*, and other *Shigella* species stratified by sex **(A)** and age group **(B)** among diarrheic under-five children in public health facilities in Debre Markos town, Northwest Ethiopia, 2022.

The frequency of *Salmonella* and *Shigella* was high among females and age group of 13–36 months as shown in Figure 2.

### Antimicrobial resistance pattern of the isolates

Both *Salmonella* serovars and *Shigella* species showed different resistance patterns against the tested antimicrobial disks. Among the tested *Salmonella* serovars, a higher rate of susceptibility was observed for Ciprofloxacin and Ceftriaxone (71.4%, 5/7 for each). In contrast, these species showed a higher rate of resistance (85.7%, 6/7) for both Ampicillin and Amoxicillin/Clavulanic acid. *S. dysenteriae* isolates were highly susceptible (87.5%, 7/8) to Ceftazidime, Ciprofloxacin, and ceftriaxone. However, they were highly resistant to Ampicillin (87.5%, 7/8) and Amoxicillin/Clavulanic acid (75%, 6/8). Other *Shigella* species showed a higher rate of susceptibility for Ciprofloxacin (90.9%, 10/11) and Ceftriaxone (81.8%, 9/11) whereas they exhibited a higher resistance rate for Amoxicillin/Clavulanic acid and Doxycycline (81.8%, 9/11 for each). All *Shigella* species showed a higher resistance pattern to Amoxicillin/Clavulanic acid (78.9%, 15/19) and Ampicillin (73.7%, 14/19) as shown in Table 4.

**Table 4:** Antimicrobial resistance pattern of *Salmonella* serovars*, S. dysenteriae*, other *Shigella* species, and all *Shigella* species isolated from under-five diarrheic children in public health facilities in Debre Markos town, Northwest Ethiopia, 2022.

**Name of antimicrobials**											
**Isolate**	**Susceptibility**	**AMP**	**AMC**	**CAZ**	**CRO**	**CIP**	**SXT**	**AZM**	**CHL**	**DOX**	**TCY**
Other *Shigella* species (*n* = 11)	*n* (%) R	7 (63.6)	9 (81.8)	2 (18.2)	1 (9.1)	0 (0)	6 (54.6)	6 (54.6)	1 (9.1)	9 (81.8)	8 (72.7)
	*n* (%) I	3 (27.3)	1 (9.1)	1 (9.1)	1 (9.1)	1 (9.1)	1 (9.1)	1 (9.1)	2 (18.2)	2 (18.2)	1 (9.1)
	*n* (%) S	1 (9.1)	1 (9.1)	8 (72.7)	9 (81.8)	9 (90.9)	4 (36.4)	4 (36.4)	8 (72.7)	0 (0)	2 (18.2)
*Shigella dysenteriae* (*n* = 8)	*n* (%) R	7 (87.5)	6 (75%)	1 (12.5)	0 (0)	0 (0)	5 (62.5)	2 (25)	2 (25)	1 (12.5)	5 (62.5)
	*n* (%) I	1 (12.5)	1 (12.5)	0 (0)	1 (12.5)	1 (12.5)	2 (25)	1 (12.5)	0 (0)	2 (25)	1 (12.5)
	*n* (%) S	0 (0)	1 (12.5)	7 (87.5)	7 (87.5)	7 (87.5)	1 (12.5)	5 (62.5)	6 (75)	5 (62.5)	2 (25)
All *Shigella* species (*n* = 19)	*n* (%) R	14 (73.7)	15 (78.9)	3 (15.8)	1 (5.3)	0 (0)	11 (57.9)	8 (42.1)	3 (15.8)	10 (52.6)	13 (68.4)
	*n* (%) I	4 (21.1)	2 (10.5)	1 (5.3)	2 (10.5)	2 (10.5)	3 (15.8)	2 (10.5)	2 (10.5)	4 (21.1)	2 (10.5)
	*n* (%) S	1 (5.3)	2 (10.5)	15 (78.9)	16 (84.2)	17 (89.5)	5 (26.3)	9 (47.4)	14 (73.7)	5 (26.3)	4 (21.1)
*Salmonella* serovars (*n* = 7)	*n* (%) R	6 (85.7)	6 (85.7)	2 (28.6)	2 (28.6)	0 (0)	5 (71.4)	3 (42.9)	5 (71.4)	2 (28.6)	5 (71.4)
	*n* (%) I	0 (0)	0 (0)	1 (14.3)	0 (0)	2 (28.6)	1 (14.3)	2 (28.6)	1 (14.3)	1 (14.3)	1 (14.3)
	*n* (%) S	1 (14.3)	1 (14.3)	4 (57.1)	5 (71.4)	5 (71.4)	1 (14.3)	2 (28.6)	1 (14.3)	4 (57.1)	1 (14.3)

R, Resistant; I, Intermediate; S, Susceptible; AMP, Ampicillin; AMC, Amoxicillin/Clavulanic acid; CAZ, Ceftazidime; CRO, Ceftriaxone; CIP, Ciprofloxacin; SXT, Trimethoprim/Sulfamethoxazole; AZM, Azithromycin; CHL, Chloramphenicol; DOX, Doxycycline; TCY, Tetracycline.

Multiple antibiotic resistance (MAR) index was calculated by dividing the number of antibiotics the isolates was resistant to the number of antibiotics the isolate was exposed to.

### Multidrug resistance pattern of the isolates

Of the culture-confirmed *Salmonella* serovars, 42.9% (3/7) were resistant to three classes of antimicrobials while nearly one-fourth (28.6% 2/7) were resistant to seven drug classes. All isolates of *Salmonella* serovars were MDR. Three-fourth [75% (6/8), 95% CI: 69.3–80.7] of culture-confirmed *S. dysenteriae* isolates and 90.9% (9/11; 95% CI: 87.1–94.7) of culture-confirmed other *Shigella* species were MDR. Moreover, 84.2% (16/19; 95% CI: 67.8–99.9) of culture-confirmed *Shigella* isolates were MDR. Overall, the rate of MDR among *Salmonella* and *Shigella* isolates was 88.5% (23/26; 95% CI: 84.3–92.7; Table 5).

**Table 5:** MDR (resistant drugs combination) pattern of *Salmonella* and *Shigella* isolates from under-five diarrheic children in public health facilities in Debre Markos town, Northwest Ethiopia, 2022.

**Antimicrobials**	**No of antimicrobial classes non-susceptible**	***Salmonella* serovars *n* (%)**	***Shigella dysenteriae n* (%)**	**Other *Shigella* Spps. *N* (%)**	**All *Shigella* Spps. *N* (%)**	**All *Salmonella* serovars and *Shigella* isolates *n* (%)**
AMC and AMP	2	-	2 (25%)	-	2 (10.5%)	2 (7.7%)
AZM, DOX, and TCY		-	-	1 (9.1%)	1 (5.3%)	1 (3.8%)
AMC, AMP, and DOX	3	1 (14.3%)	-	1 (9.1%)	1 (5.3%)	2 (7.7%)
AMC, AMP, and SXT		1 (14.3%)	1 (12.5%)	1 (9.1%)	2 (10.5%)	3 (11.6%)
AMC, AZM, and SXT		-	-	1 (9.1%)	1 (5.3%)	1 (3.8%)
AZM, CHL, and TCY		1 (14.3%)	-	-	-	1 (3.8%)
AMC, TCY, and SXT		-	1 (12.5%)	-	1 (5.3%)	1 (3.8%)
AMC, DOX, TCY, and SXT		-	-	1 (12.5%)	1 (5.3%)	1 (3.8%)
AMC, AMP, CHL, and TCY	4	-	1 (12.5%)	-	1 (5.3%)	1 (3.8%)
AMC, AMP, AZM, DOX, and TCY		-	-	2 (18.2%)	2 (10.5%)	2 (7.7%)
AMC, AMP, DOX, TCY, and SXT		-	1 (12.5%)	-	1 (5.3%)	1 (3.8%)
AMP, AZM, CHL, DOX, and TCY		-	-	1 (9.1%)	1 (5.3%)	1 (3.8%)
AMC, CAZ, CRO, DOX, TCY, and SXT		-	-	1 (9.1%)	1 (5.3%)	1 (3.8%)
AMC, AMP, CHL, TCY, and SXT	5	1 (14.3%)	-	-	-	1 (3.8%)
AMP, AZM, CAZ, TCY, and SXT		-	1 (12.5%)	-	1 (5.3%)	1 (3.8%)
AMP, AZM, CHL, TCY, and SXT		-	1 (12.5%)	-	1 (5.3%)	1 (3.8%)
AMC, AMP, AZM, DOX, TCY, and SXT		-	-	1 (9.1%)	1 (5.3%)	1 (3.8%)
AMC, AMP, CAZ, DOX, TCY, and SXT		-	-	1 (9.1%)	1 (5.3%)	1 (3.8%)
AMC AMP, CHL, DOX, TCY, and SXT		1 (14.3%)	-	-	-	1 (3.8%)
AMC AMP, AZM, CAZ, CRO, CHL, TCY, and SXT	7	2 (28.6%)	-	-	-	2 (7.7%)
Total		7 (100%)	8 (100%)	11 (100%)	19 (100%)	26 (100%)

R, Resistant; I, Intermediate; S, Susceptible; AMP, Ampicillin; AMC, Amoxicillin/Clavulanic acid; CAZ, Ceftazidime; CRO, Ceftriaxone; CIP, Ciprofloxacin; SXT, Trimethoprim/Sulfamethoxazole; AZM, Azithromycin; CHL, Chloramphenicol; DOX, Doxycycline; TCY, Tetracycline.

### Factors associated with the prevalence of *Salmonella* and *Shigella*

After adjusting for confounding factors, parents'/guardians' educational level ≤ elementary school, presence of two or more under-five children in the family, unimproved source of drinking water, a frequent habit of storing cooked food for later use, attendance of the child at social gatherings within 1 week before infection, and infrequent child fingernail trimming (≥2 weeks) were significant predictors of *Salmonella* and *Shigella* among under-five diarrheic children (*P* ≤ 0.05; Table 6).

**Table 6:** Bivariable and multivariable logistic regression analysis of factors associated with *Salmonella* and *Shigella* infection among under-five children at public health facilities in Debre Markos town, 2022.

**Variable**	***Salmonella*** **or** ***Shigella*** **infection**		**COR (95% CL)**	***P*-value**	**AOR (95% CL)**	***P*-value**
	**Yes (%)**	**No (%)**				
**Child sex**						
Male	11 (42.3)	106 (54.1)	0.623 (0.272, 1.424)	0.262	-	-
Female	15 (57.7)	90 (45.9)	1			
**Child age (in months)**						
37–59	8 (30.8)	82 (41.8)	0.663 (0.202, 2.174)	0.498	-	-
13–36	13 (50.0)	80 (40.8)	1.105 (0.365, 3.342)	0.860		
6–12	5 (19.2)	34 (17.3)	1			
**Attendance of the child at daycare (KG)**						
Yes	5 (19.2)	58 (29.6)	0.567 (0.204, 1.575)	0.276	-	-
No	21 (80.8)	138 (70.4)	1			
**Parent/guardian age (in years)**						
< 29	6 (23.1)	93 (47.4)	0.335 (0.95, 1.187)	0.090^+^		0.806
30–39	15 (57.7)	77 (39.3)	1.013 (0.335, 3.060)	0.982		
>40	5 (19.2)	26 (13.3)	1			
**Parent/guardian educational level**						
Elementary and below	15 (57.7)	36 (18.4)	6.061 (2.570, 14.293)	< 0.0001^+^	3.78 (1.28, 11.19)	0.016^++^
Secondary and above	11 (42.3)	160 (81.6)	1		1	
**Number of under-five children in the family**						
Two and above	15 (57.7)	26 (13.3)	8.916 (3.696, 21.511)	< 0.0001^+^	8.999 (2.92, 27.69)	< 0.0001^++^
One	11 (42.3)	170 (86.7)	1		1	
**Parent/guardian occupation**						
civil servant	8 (30.8)	79 (40.3)	0.871 (0.268, 2.827)	0.818	-	-
Self-employed	13 (50.0)	74 (37.8)	1.511 (0.504, 4.528)	0.461		
Housewife	5 (19.2)	43 (21.9)	1			
**Family size in the household**						
5–7	17 (65.4)	67 (34.2)	3.637 (1.539, 8.596)	0.003^+^	2.56 (0.78, 8.46)	0.122
2–4	9 (34.6)	129 (65.8)	1			
**Child's history of diarrhea in the last month**						
Yes	5 (19.2)	40 (20.4)	0.929 (0.330, 2.615)	0.888	-	-
No	21 (80.8)	156 (79.6)	1			
**Child nutritional status**						
Under-nutrition	19 (73.1)	79 (40.3)	4.020 (1.614, 10.010)	0.003^+^	1.09 (0.32, 3.70)	0.888
Normal	7 (26.9)	117 (59.7)	1			
**House floor material**						
Poor	6 (23.1)	8 (4.1)	7.050 (2.222, 22.366)	0.001^+^	1.36 (0.20, 9.45)	0.755
Good	20 (76.9)	188 (95.9)	1			
**Hand washing facility near the latrine**						
No	18 (69.2)	90 (45.9)	2.650 (1.100, 6.382)	0.030^+^	1.67 (0.53, 5.23)	0.379
Yes	8 (30.8)	106 (54.1)	1			
**Parent/guardian hand washing habit after toilet**						
Sometimes or no	8 (30.8)	36 (18.4)	1.975 (0.797, 4.897)	0.142^+^	0.92 (0.22, 3.82)	0.907
Usually or always	18 (69.2)	160 (81.6)	1			
**Presence of a separate waste disposal system**						
No	17 (65.4)	69 (35.2)	3.477 (1.472, 8.212)	0.004^+^	1.68 (0.52, 5.42)	0.386
Yes	9 (34.6)	127 (64.8)	1			
**The habit of washing a child's hands after child defecation**						
Sometimes or no	9 (34.6)	43 (21.9)	1.884 (0.785, 4.523)	0.156^+^	0.94 (0.26, 3.36)	0.926
Usually or always	17 (65.4)	153 (78.1)	1			
**Hand-washing habit before feeding the child**						
Sometimes or no	9 (34.6)	21 (10.7)	4.412 (1.747, 11.138)	0.002^+^	2.58 (0.54, 12.37)	0.238
Usually or always	17 (65.4)	175 (89.3)	1			
**Source of drinking water**						
Unimproved	14 (53.8)	48 (24.5)	3.597 (1.558, 8.307)	0.003^+^	5.01 (1.56, 16.10)	0.007^++^
Improved	12 (46.2)	148 (75.5)	1		1	
**A habit of storing cooked food for later use**						
Usually or always	15 (57.7)	45 (23.0)	4.576 (1.963, 10.666)	< 0.0001^+^	3.199 (1.07, 9.54)	0.037^++^
Sometimes or no	11 (42.3)	151 (77.0)	1		1	
**Attendance at social gatherings in the past week**						
Yes	17 (65.4)	57 (29.1)	4.606 (1.940, 10.937)	< 0.0001^+^	5.387 (1.78, 16.35)	0.003^++^
No	9 (34.6)	139 (70.9)	1		1	
**Travel history of the child in the past week**						
Yes	5 (19.2)	37 (18.9)	1.023 (0.362, 2.891)	0.966	-	-
No	21 (80.8)	159 (81.1)	1			
**The time interval for fingernail trimming**						
Two weeks and above	15 (57.7)	57 (29.1)	3.325 (1.440, 7.679)	0.005^+^	4.693 (1.47, 14.94)	0.009^++^
Below 2 weeks	11 (42.3)	139 (70.9)	1		1	

COR, crude odds ratio; AOR, adjusted odds ratio; CI, confidence interval.

+ Variables with P ≤ 0.25.

++ Variables with P ≤ 0.05.

1, reference category.

Children whose parent/guardian had below primary school educational level have about four times more likely to get *Salmonella* and *Shigella* infection compared to their counterparts (AOR = 3.78; 95% CI = 1.28–11.19; *P* = 0.016). It is about nine times (AOR = 8.999; 95% CI = 2.93–27.69; *P* = 0.000) more likely that a child who had been living in a family with two or more under-five children was infected with *Salmonella* and *Shigella*. Children who drank unimproved water were five times more likely to get infected with *Salmonella* and *Shigella* than those who drank improved water (AOR = 5.010; 95% CI = 1.56–16.098; *P* = 0.007).

Children with parents/guardians with the habit of storing cooked food for later use were about 3.2 times in odds of getting an infection with *Salmonella* and *Shigella* compared to their counterparts (AOR = 3.199; 95% CI = 1.07–9.54; *P* = 0.037). Children who had attended social gatherings had about five times chance of harboring *Salmonella* and *Shigella* (AOR = 5.387; 95% CI = 1.78–16.35; *P* = 0.003). Besides, infrequent child fingernail trimming was another independent predictor of Salmonella and *Shigella* infection (AOR = 4.693; 95% CI = 1.47–14.94; *P* = 0.009; Table 6).

## Discussion

*Enterobacteriaceae* are among the predominant causative agents of gastroenteritis and *Salmonella* serovars and *Shigella* species are among the foremost agents. In this study, the overall prevalence of culture-confirmed *Salmonella* and *Shigella* infection among under-five diarrheic children was 11.7% (26/222; 95% CI: 7.2–15.5). This result is in line with studies conducted in different parts of Ethiopia; Dire Dawa 9.2% (13), Hawassa 9.5% (12), Bale Robe 11.2% (19), and Hosanna 9.5% (17). It is also in line with studies conducted in different countries like Kenya 12.5% (38), Zambia 12.4% (39), and Bangladesh 8.5% (40). Moreover, it is also in agreement with a systematic review and meta-analysis done in Sub-Saharan Africa 14.1% (23). However, it is lower than studies conducted in Arba Minch-Ethiopia 17.45% (18), Zanzibar 28.84% (41), India 36.4% (42), Iran 15.9% (43), and another study in Iran 25.9% (44). The reason for this difference could be due to the difference in the number of study participants enrolled. The number of study participants enrolled in previous studies was higher compared to our study. Moreover, the duration of the study period may also be another factor since these studies were conducted over a relatively longer study period (up to 4 years). The result of this study is higher than studies reported from Ambo-Ethiopia 3.8% (16) to Sudan 6% (45). This discrepancy could be due to the difference in study time which is 2014 for a study from Ambo-Ethiopia and 2013 for the study from Sudan. Moreover, seasonal variation may also be another factor. The study conducted in Ambo was conducted in winter while this study was conducted in summer.

The prevalence of culture-confirmed *Salmonella* serovars was 3.2% (95% CI: 0.9–5.6%) which is comparable with studies from different parts of Ethiopia; Debre Berhan 3.1% (46), Addis Ababa 3.95% (11), Dire Dawa 3.6% (13), Hawassa 2.5% (12) and different countries like Sudan 2% (45), and Bangladesh 4.9% (40). It is also comparable with a meta-analysis done in Sub-Saharan Africa 4.5% (23) and the global prevalence of 1.9% (47). This finding is lower than results reported from studies conducted in other parts of Ethiopia; Bahir Dar 7.8% (26), Bale Robe 6.9% (19), Arba Minch 12.6% (18), and different countries such as Zambia 8% (39), India 20.5% (42), Iran 13.1% (44), and another study in Iran 7% (43). These differences could be associated with differences in geographical location, and study populations study participants in Iran and India were selected from admission rooms in selected hospitals. This finding is slightly higher than reports from different areas of Ethiopia; Ambo 1.3% (16) and Hosanna 1% (17) which could be due to difference in study time as studies from Ambo and Hosanna were conducted in 2013 and 2017, respectively.

The prevalence of culture-confirmed *Shigella* species was 8.6% (95% CI: 4.9–12.3%) which is comparable with reports from different areas of Ethiopia; Bahir Dar 9.5% (26), Addis Ababa 9.1% (11), Dire Dawa 5.6% (13), Hawassa 7% (12), and Hosanna 8.3% (17). It is also in agreement with studies in different countries; Kenya 7.4% (48), another study from Kenya 5% (38), Mexico 6% (49), and Iran 8.9% (43). Moreover, it is also in line with a meta-analysis done in Sub-Saharan Africa 9.6% (23). Our finding is lower than studies reported from Zanzibar 16.93% (41), India 15.9% (42), Iran 26.17% (50), and another study from Iran 12.8% (44). This variation could be due to differences in the study setting and sampling technique. In these studies, outpatients and admitted patients were selected from different hospitals. A systematic random sampling technique was used in these studies to select participants. Moreover, the number of participants enrolled was relatively larger compared to our study. In contrast to this study, a lower rate of isolation was reported from parts of Ethiopia; Debre Berhan 1.8% (46), Ambo 2.5% (16), Bale Robe 4.3% (19), Arba Minch 4.85% (18) and different countries; Sudan 4% (45), Zambia 4.4% (39), and Bangladesh 3.6% (40). The reason for this difference could be seasonal variations and variations in socio-demographic factors. Studies in Ambo, Bale Robe, Arba Minch, and Debre Berhan were conducted in the winter season, but our study was conducted in the summer.

The antimicrobial resistance rate of culture-confirmed *Salmonella* serovars and *Shigella* species for different antimicrobials has been increasing around the world, especially in developing countries. In our study, 10 antimicrobials from eight different drug classes were tested for isolates showing different rates of resistance. A high level of resistance of *Salmonella* isolates was observed for ampicillin [%R = 85.7% (6/7), 95% CI: 42–99.2%]. This is in line with previous studies conducted in different parts of Ethiopia; Bahir Dar 93.9% (26), Addis Ababa 80% (11), Ambo 88.9% (16), and Dire Dawa 71.4% (13). However, it is lower than studies conducted in Debre Markos 100% (51), Alamura 100% (20), Hosanna 100% (17), and Arba Minch 100% (18). This could be due to differences in the study population. A study in Debre Markos was done among food handlers and a study conducted in Alamura was carried out on under 14 children. *Salmonella* isolates demonstrated a high rate of resistance to Chloramphenicol [71.4% (5/7), 95% CI: 30.3–94.9]. This is in line with a study from Arba Minch 43% (18) but higher than studies from other parts of Ethiopia; Dire Dawa 14.3% (13), Bale Robe 24.1% (19), and Hosanna 0% (17). This could be due to the increasing habit of using antimicrobials in cattle production in the country.

Our study revealed that isolated *Shigella* species had shown a high rate of resistance to amoxicillin/clavulanic acid 78.9% (15/19; 95% CI: 60.55–97.25). This is in line with a study conducted in Addis Ababa 91.4% (11). In contrast, it is higher than reports from Gondar 10.3% (28), Alamura 55.6% (20), and French Guiana 12% (52). In our study, the resistance rate of *Shigella* species for ampicillin was 73.7% (14/19; 95% CI: 53.9–93.5). This finding is comparable with reports from Ethiopia; Gondar 76% (28), Hawassa 63.6% (12), Alamura 81.8% (20), Hosanna 82.4% (17), Ambo 88.9% (16), Dire Dawa 90.9% (13), and also different countries such as French Guiana 58% (52), Iran 67.8% (44), and another study in Iran 61.5% (53). Moreover, it is also in line with the result of pooled Ethiopian studies 83.1% (54). On the contrary, it is lower than reports from Debre Markos 100% (51), Addis Ababa 95.7% (11), and Iran 96.2% (43). This variation could be due to differences in study time, study population, and socio-demographic factors.

In our study, 100% (7/7) of *Salmonella* isolates were MDR. This is higher than reports from different parts of Ethiopia; Bale Robe (31.03%) (19), Arba Minch (37.5%) (18), Hosanna (50%) (17), Ambo (66.7%) (16), and in Bangladesh (26.67%) (40). Moreover, 84.2% (16/19; 95% CI: 67.8–99.9) of culture-confirmed *Shigella* isolates were MDR. This result is in line with reports from Ambo-Ethiopia (83.3%) (16), Iran (92%) (55), and Bangladesh (93%) (40). Howe ever, this is higher than reports from different parts of Ethiopia; Gondar (37.9%) (28), Arba Minch (37.5%) (18), Hosanna (64.7%) (17), and in Nigeria (58.3%) (43). This discrepancy could be due to the widespread nature of resistant strains, the emergence of new resistant strains, and the increasing habit of using antimicrobials in cattle and poultry production. Moreover, an increasing habit of antimicrobial misuse with self-prescription may also have a contribution.

In this study, children whose parents/guardians' educational level ≤ elementary school had higher odds of getting infection with *Salmonella* and *Shigella*. This may be due to a low level of knowledge, attitude, and practice as compared to parents/guardians with secondary school and above educational level. The presence of two or more under-five children in a family had increased the risk of getting *Salmonella* and *Shigella* infections in 9-folds. This result is supported by a study conducted in Cameroon (56). This may be due to the inability of parents/guardians to provide appropriate care for those children because of cost and time shortages.

In our study, the habit of storing cooked food for later use had a significant association with the prevalence of *Salmonella* and *Shigella* which is supported by a study conducted in Alamura (southern Ethiopia) (20). This could be because bacteria can multiply in significant numbers and cause disease upon storage in ambient temperatures. Similarly, infrequent fingernail trimming (≥2 weeks) had increased the odds of getting an infection with *Salmonella* and *Shigella* by 4.7 times. This result is in agreement with a study done in Arba Minch (18) entailinguntrimmed fingers can easily be contaminated with soil and other wastes carrying the indicated bacteria.

This study confirmed that participants who had unimproved sources of drinking water have higher odds (five times) of getting infected with *Salmonella* and *Shigella* comparable with a study conducted in Dire Dawa (13). It is known that contaminated water and food are responsible for the transmission of diarrheal diseases. Other factors associated with a high rate of *Salmonella* and *Shigella* in under-five diarrheic children was the attendance of the child at social gatherings within 1 week before disease onset. This increased the risk of infection by 5.4 times. This may be because attending and eating at these ceremonies may increase the risk of contamination and infection.

In this study, we couldn't identify *Salmonella* serovars and all *Shigella* species and also failed to include molecular techniques which could hamper the anticipated prevalence of the bacterial isolates. Having these limitations, this study provides a glimpse into the prevention and control of drug resistance and diarrhea caused by *Salmonella* and *Shigella* in the study area.

## Conclusion

In this study, the prevalence of *Salmonella* and *Shigella* infection was significantly high compared to the global prevalence. Most *Shigella* isolates were highly resistant to amoxicillin/clavulanic acid, doxycycline, and tetracycline whereas most *Salmonella* isolates were highly resistant to ampicillin, amoxicillin/clavulanic acid, trimethoprim-sulfamethoxazole, chloramphenicol, and tetracycline. Both *Salmonella* and *Shigella* isolates exhibited a high rate of MDR pattern. Ceftazidime, ceftriaxone, ciprofloxacin, and chloramphenicol could be possible antimicrobial choices in case of *Shigella* infection while ceftazidime and ciprofloxacin could be antimicrobials of choice for treating *Salmonella* infection. Our study also revealed that low parent/guardian educational level, presence of two or more under-five children in the family, consumption of unimproved water, the habit of storing cooked foods for later use, attendance of the child at social gatherings, and infrequent fingernail trimming were independent predisposing factors for the higher rate of *Salmonella* and *Shigella* infection. Culture-based bacterial detection and antimicrobial sensitivity test-based antimicrobial prescription should be routinely practiced. Public health measures should be in place to improve the hygiene of the community, improve sources of drinking water, create awareness in the community, and improve environmental and personal hygiene. Large-scale longitudinal studies are also recommended.

## Data availability statement

The original contributions presented in the study are included in the article/supplementary material, further inquiries can be directed to the corresponding author.

## Ethics statement

Ethical clearance was obtained from Debre Markos University College of Health Sciences before the actual data collection with letter number HSC/R/C/Ser/PG/Co/195/11/14 and Amhara Public Health Institute with a letter numbered 03/1434. Written informed consent to participate in this study was provided by the participants' legal guardian/next of kin.

## Author contributions

MD conceived and designed the study, performed the data collection and analysis, and wrote the manuscript. HM and GM supervised and assisted in the designing, data analysis and interpretation, and critical editing and revision of this manuscript. All authors read and approved the final manuscript.
